# Evaluation of amniotic mesenchymal cell derivatives on cytokine production in equine alveolar macrophages: an in vitro approach to lung inflammation

**DOI:** 10.1186/s13287-016-0398-9

**Published:** 2016-09-20

**Authors:** Enrica Zucca, Emanuela Corsini, Valentina Galbiati, Anna Lange-Consiglio, Francesco Ferrucci

**Affiliations:** 1 Department of Health, Animal Science and Food Safety (VESPA), Università degli Studi di Milano, Via Celoria 10, 20133 Milano, Italy; 2 Laboratory of Toxicology, DiSFeB, Università degli Studi di Milano, Milan, Italy; 3 Large Animal Hospital, Reproduction Unit, Università degli Studi di Milano, Lodi, Italy

**Keywords:** Horse, Amniotic mesenchymal cell derivatives, Alveolar macrophages, Cytokine

## Abstract

**Background:**

Data obtained in both animal models and clinical trials suggest that cell-based therapies represent a potential therapeutic strategy for lung repair and remodeling. Recently, new therapeutic approaches based on the use of stem cell derivatives (e.g., conditioned medium (CM) and microvesicles (MVs)) to regenerate tissues and improve their functions were proposed. The aim of this study was to investigate the immunomodulatory effects of equine amniotic mesenchymal cell derivatives on lipopolysaccharide (LPS)-induced cytokine production in equine alveolar macrophages, which may be beneficial in lung inflammatory disorders such as recurrent airway obstruction (RAO) in horses. RAO shares many features with human asthma, including an increased number of cells expressing mRNA for interleukin (IL)-4 and IL-5 and a decreased expression of IFN-γ in bronchoalveolar lavage fluid (BALF) of affected horses.

**Methods:**

The release of TNF-α, IL-6, and TGF-β1 at different time points (1, 24, 48, and 72 h) was measured in equine alveolar macrophages stimulated or not with LPS (10 and 100 ng/mL) in the presence or absence of 10 % CM or 50 × 10^6^ MVs/mL.

Cytokines were measured using commercially available ELISA kits. For multiple comparisons, analysis of variance was used with Tukey post-hoc test. Differences were considered significant at *p* ≤ 0.05.

**Results:**

Significant modulatory effects of CM on LPS-induced TNF-α release at 24 h, and of both CM and MVs on TNF-α release at 48 h were observed. A trend toward a modulatory effect of both CM and MVs on the release of TGF-β and possibly IL-6 was visible over time.

**Conclusions:**

Results support the potential use of CM and MVs in lung regenerative medicine, especially in situations in which TGF-β may be detrimental, such as respiratory allergy. Further studies should evaluate the potential clinical applications of CM and MVs in equine lung diseases, such as RAO and other inflammatory disorders.

## Background

Regenerative medicine is a new therapeutic approach based on the use of stem cells and their derivatives (e.g. conditioned medium (CM) and microvesicles (MVs)) to regenerate tissues and to improve their function, including lung diseases [1–5].

Several studies have demonstrated that both mouse and human embryonic stem cells can be induced in culture to acquire phenotypic markers of type 2 alveolar epithelial cells, including expression of surfactant proteins and lamellar bodies, and formation of pseudoglandular structures. However, there are only limited available studies concerning the effects of embryonic stem cells, or of their derivatives, on in vivo lung pathologies [4, 5].

In parallel, other studies speculated that adult bone marrow-derived or cord blood-derived stem cells could engraft and differentiate in mature airway and alveolar epithelial cells, vascular endothelial cells, or interstitial lung cells. Interestingly, several recent reports suggested that both systemic and intrapulmonary administration of bone marrow-derived mature stem cells in mice models of acute lung injury resulted in decreased mortality, improved alveolar fluid clearance, and attenuated inflammation and lung injury, despite minimal, if any, stem cell engraftment in the lung [1]. To explain the action of mesenchymal stem cells (MSCs), several paracrine mechanisms have been proposed for bone marrow-derived stem cell effects, including release of anti-inflammatory mediators such as interleukin (IL)-10, angiopoietin-1, and keratinocyte growth factor (KGF) that could modulate immune responses.

Researchers are considering other sources of stem cells for airway tissue engineering. Extra-fetal-derived stem cells could represent new alternative sources for lung tissue regeneration. Indeed, recently, amniotic mesenchymal cells (AMCs) isolated from human and horse term placenta showed many characteristics of stem cells including a very low antigenicity, no tumorigenic effects, and the potential to differentiate into mesodermal and ectodermal lines [6–10]. Moreover, the therapeutic use of both equine AMCs and CM in tendon and ligament injuries in vivo showed that the regenerative property of stem cells is probably due either to their differentiation capacity or to production of mediators capable of activating the intrinsic reparative processes of damaged tissues [11]. In addition to soluble factors present in CM, recent studies showed that stem cells could communicate with target cells through paracrine mechanism by means of MVs [12, 13].

Among MVs, two different populations could be identified: exosomes, which originate from invaginations of the endosome membranes and which have a diameter between 30 and 120 nm, and the shedding vesicles that are more heterogeneous in size (80 nm to 1 micron) and arise directly by protrusions of the cell membrane. MVs express different surface markers, including binding receptors to the target cell surface. When such link occurs, the microvesicle membrane merges with that of the target cell releasing its content into the cell cytoplasm [14–16]. It was demonstrated that MVs could be employed in vitro and in vivo for tissue repair, increasing the degree of healing [17–19]. In line with these findings, new studies proposed the use of CM or purified MVs for regenerative treatment of damaged tissues in place of stem cells.

Among equine respiratory disorders, recurrent airway obstruction (RAO), due to its clinical aspects, is similar to some kinds of human asthma suggesting a common immunological basis [20]. RAO, reported as a debilitating and incurable respiratory disease affecting stabled mature horses worldwide, shares many features with human asthma, including lower airway inflammation, reversible airflow obstruction, bronchial hyper-responsiveness, mucus accumulation, and remodeling, resulting in severe clinical signs [21, 22]. Common clinical signs include coughing, respiratory distress, and increased breathing effort during periods of exacerbation, which are triggered by hypersensitivity reactions to allergens and irritants mainly from hay dust. Moreover, RAO and human severe asthma share many structural changes such as epithelial detachment and regeneration, goblet cell hyperplasia, and hyperplasia of the bronchial smooth muscles, probably related to neutrophils activity [20–22]. While the immunogenetic background of RAO is still not completely understood, several studies have shown that interactions of innate and adaptive immune responses play an important role [21]. Finally, an increased number of cells expressing mRNA for IL-4 and IL-5 and a decreased expression of IFN-γ was demonstrated in bronchoalveolar lavage fluid (BALf) of RAO affected horses [23]. Some studies showed contradictory results regarding the involvement of cytokines characteristic for the Th1 or Th2 type of immune response and it has been suggested that cytokine profiles reflecting both types of Th responses are observed at different time points after antigen challenge, similar to that demonstrated in human asthma.

RAO develops over a period of years, and offers a unique model to study the respiratory system under chronic inflammatory conditions [22]. To date, similar to asthma, RAO can be controlled by the administration of corticosteroids and bronchodilators.

The aim of the present study was to evaluate the in vitro effect of AMC derivatives (CM and MVs) on the production of pro- and anti-inflammatory cytokines (tumor necrosis factor (TNF)-α, IL-6, and transforming growth factor (TGF)-β) in equine alveolar macrophages (AMs) collected by means of bronchoalveolar lavage (BAL), following stimulation with lipopolysaccharide (LPS). LPS was used according to Gupta et al. [1] and Mei et al. [2], who demonstrated its capability for inducing lung inflammation both in vivo and in vitro [3, 24].

The study was approved by the University of Milan Ethics Committee (Protocol Number 41/15) and informed client consent was obtained for inclusion in the study.

## Methods

Chemicals, cell culture media and supplements were obtained from Sigma-Aldrich Chemical (Milan, Italy) unless otherwise specified, and tissue culture dishes were purchased from Euroclone (Milan, Italy).

### Tissue collection and cell isolation

All procedures to collect allanto-amniotic membrane were conducted following standard veterinary practice and in accordance with the 2010/63 EU directive on animal protection and Italian Law (D.L. No. 116/1992). Allanto-amniotic membranes were obtained at term from normal pregnancies of three mares and amniotic cells were obtained as described elsewhere [10]. Amniotic cells were cultured and expanded to passage (P)3 to obtain CM or MVs.

### Preparation of CM

CM was obtained as previously described [11], pooling the media obtained from the different amniotic samples. Briefly, AMCs at P3 were plated into 24-well plates at a density of 5 × 10^4^ cells/ml/well in serum-free Dulbecco’s modified Eagle’s medium (HG-DMEM). To generate AMC-CM, cells were cultured for 5 days at 38.5 °C in a humidified atmosphere of 5 % CO_2_. Supernatants from each plate were then collected, pooled, centrifuged at 2500 g, and stored at –20 °C until use.

### Isolation and measurement of MVs

MVs were obtained from the culture media of AMCs derived from three different placentas, cultured for a week with HG-DMEM supplemented with 10 % MV-deprived fetal calf serum (FCS) and overnight in HG-DMEM deprived of FCS and supplemented with 0.5 % BSA. The isolation and measurement of MVs were performed as previously described [19]. Briefly, the overnight culture media were centrifuged at 2000 g for 20 min to remove debris, then at 100,000 g (Beckman Coulter Optima L-100 K ultracentrifuge) for 1 h at 4 °C, washed in serum-free medium 199 containing N-2-hydroxyethylpiperazine-N-2-ethanesulfonic acid (HEPES) 25 mM and submitted to a second ultracentrifugation under the same conditions. The pellet was immediately resuspended in HG-DMEM, and a sample of the resuspended pellet was taken for measurement of MV size and concentration. The remaining part of the pellet was cryopreserved with 1 % of dimethylsulfoxide at –80 °C and used for the in vitro test. Size and concentration of MVs were evaluated by the Nanosight LM10 instrument (Nanoparticle tracking analysis, NTA, Nano-Sight Ltd., Amesbuty, UK), which permits discrimination of microparticles less than 1 μm in diameter. The software (NTA 2.0 analytic software) allows the analysis of video images of particle movement under Brownian motion and the calculation of diffusion coefficient, sphere equivalent, and hydrodynamic radius of particles by using the Strokes–Einstein equation.

### Bronchoalveolar lavage fluid

BALf was obtained as previously described [25, 26] from horses admitted to the LAHUM (Large Animal Hospital, University of Milan) to undergo a specific diagnostic protocol for poor performance, including airways endoscopy and BAL. Part of the BALf, after execution of diagnostic procedures, was used for the in vitro study. For cytology, an aliquot of BALf, placed in tubes with EDTA, was centrifuged (Cytospin Rotofix 32, Hettich, Tuttlingen, Germany) at 25 g for 5 min and stained with May Grünwald Giemsa for total and differential cell counts [25, 26]. The remaining aliquot of BALf was placed in plain sterile tubes. Cells were obtained by means of centrifugation at 1200 rpm for 10 min and washed with RPMI-1640 supplemented with 2 mM l-glutamine, 0.1 mg/mL streptomycin, 100 IU/mL penicillin, and 5 μg/mL gentamicin. Cells were diluted at 2 × 10^6^/mL in the same medium. To promote AMs adhesion, 1 mL of cell suspension was placed in 24-well plates and incubated at 37 °C for 1 h. The adherent cells were subsequently washed with medium to remove the non-adherent cells. One milliliter of medium supplemented with 10 % FBS was added to each well in the presence or absence of LPS (obtained from *Escherichia coli* serotype 0127: B810) to a final concentration of 10 and 100 ng/mL, in the presence or absence of 10 % CM, or in the presence or absence of 50 × 10^6^ MVs/mL.

Cells were incubated for 1, 24, 48, and 72 h. After incubation, supernatants were collected by centrifugation to remove detached cells and stored at –20 °C in 1.5-mL tubes until the measurement of cytokines was carried out.

### Measurement of cytokines

TNF-α, IL-6, and TGF-β1 measurements were made using commercially available ELISA kits (Genorise, Glen Mills, PA, USA) according to the manufacturer’s specifications. Results are given as pg/mL or as percentage of relative controls.

### Cellular viability

In the experiments with recombinant equine TGF-β, cell viability was evaluated by the MTT test [27]. Briefly, 100 μL of cells were seeded in a 96-well plate and treated in the presence or absence of equine TGF-β (300 pg/mL). After 24-h incubation, the medium was removed and 100 μL/well of MTT solution (0.75 mg/mL) was added. Cells were incubated for 3 h at 37 °C, the medium was discarded, and cells lysed in 100 μL/well of a mixture of HCl 1N:isopropanol (1:24). The absorbance of the resulting solutions was read at a wavelength of 595 nm in a microplate reader (EMax, Molecular Devices, Sunnyvale CA, USA). Results are expressed as a percentage of control.

### Statistical analysis

Statistical analysis was performed using GraphPad InStat version 3.0a for Macintosh (GraphPad Software, San Diego, CA, USA). For multiple comparisons, analysis of variance was used with Tukey post-hoc test. Differences were considered significant at *p* ≤ 0.05. Results are presented as mean ± SEM.

## Results

### Tissue collection and cell isolation

**Fig. 1:**
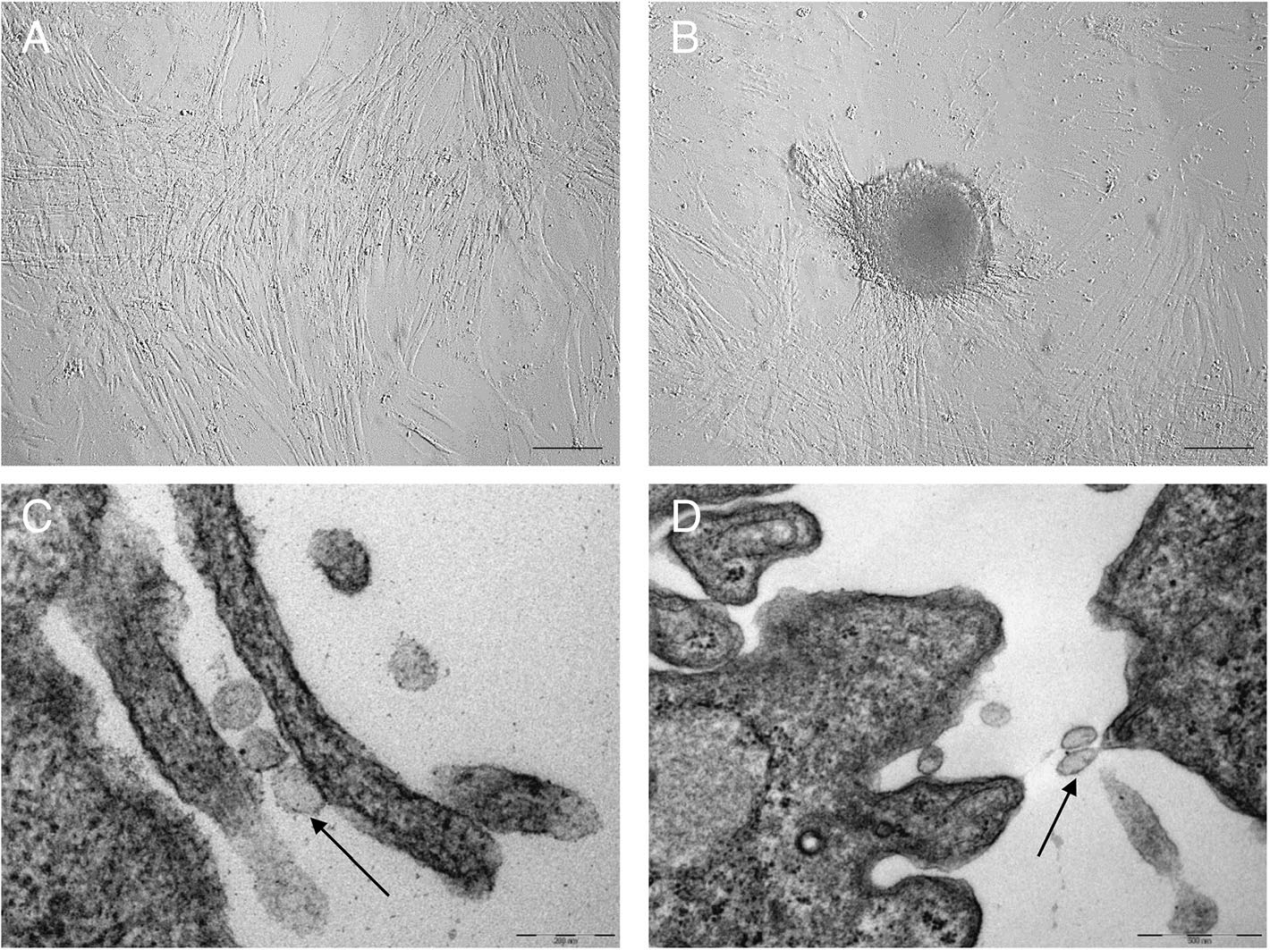
Cell morphology. **a** Monolayer of cells in first culture. **b** Fibroblast-like cells organized in cluster. *Scale bars* = 20 μm. Magnification 20×. Electron microscopy analysis of MVs; arrows in **c** and **d** show the release of MVs from the surface of AMCs. *Scale bars* = 200 nm for (**c**) and 500 nm for (**d**)

Cells were selected purely on their ability to adhere to plastic. The initial viability of AMCs was >90 %. Amniotic cells (Fig. 1a) displayed typical fibroblast-like morphology. AMCs observed at the early stages of culture were organized as three-dimensional clusters (Fig. 1b). Molecular biology analyses at P3 showed that these AMCs display a typical mesenchymal stromal phenotype, with the expression of markers such as CD29, CD44, CD106, CD105, and MHCI, but not CD34 and MHCII. Moreover, AMCs showed differentiative potential in mesenchymal (osteogenic, adipogenic, and chondrogenic) and ectodermic lines (neurogenic) as reported by Lange-Consiglio et al. [10].

### Isolation and measurement of MVs

In all samples, the viability of AMCs at the time of MV collection was 99 % as detected by Trypan blue exclusion. The size of MVs ranged from 50 nm to 670 nm, with a mean size of 258 ± 55 nm for the three samples. The number of MVs ranged from 800 to 4700 particles/cell, with a mean value of 2550 ± 71 particles/cell (corresponding to 540 × 10^6^ particles/mL of medium). From this result, 10 % of CM contains about 50 × 10^6^ MVs and this dose of MVs was used in our experiments. This dose was chosen on the base of a dose-response curve studied on others target cells where this amount corresponded to the higher uptake of labeled MVs [19].

Transmission electron microscopy analysis [19] revealed the presence of variably sized extra-cellular membranous vesicles budding from, or lying near, the cell of origin (Fig. 1c, d). The size of MVs ranged from 100 nm to 1000 nm, with a predominance of vesicles between 100 and 200 nm. Because of size and morphological characteristics, the vesicles observed were mainly considered as shedding vesicles.

### Cytology of BALf

Total and differential cell counts, reported as the mean of five samples ± SEM, are shown in Table 1. Approximately 29.2 ± 3.1 × 10^6^ AMs were isolated from each of the five horses. Cell viability on the day of harvesting was >95 % as assessed by Trypan blue exclusion.

**Table 1:** Total and differential cell counts of bronchoalveolar lavage.

	Bronchoalveolar lavage (%)*
Macrophages (%)	58.4 ± 6.2
Lymphocytes (%)	22.8 ± 3.2
Neutrophils (%)	16.2 ± 4.8
Mast cells (%)	1.2 ± 1.3
Eosinophils (%)	1.4 ± 1.9
Total cell count (cells/μL)	460 ± 21.2

*Results are the mean of five samples ± SEM

### Characterization of cytokine release in equine alveolar macrophages

**Fig. 2:**
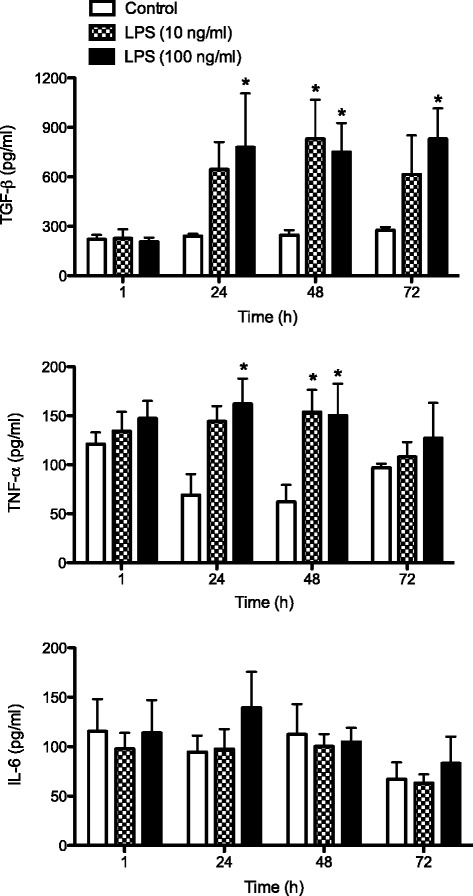
Time-course and dose-response of lipopolysaccharide (*LPS*)-induced cytokine production. Alveolar macrophages were seeded at 2 × 10^6^/mL and, after adherence, treated for 1, 24, 48, and 72 h in the presence or absence of LPS (10 and 100 ng/mL). The release of tumor necrosis factor alpha (*TNF*-*α*), interleukin-6 (*IL*-*6*), and transforming growth factor beta (*TGF*-*β*) were evaluated in conditioned medium by ELISA. Results are the mean of a minimum of four experiments ± SEM. Statistical analysis was performed by Tukey’s multiple comparison test, with *p < 0.05 versus control cells

AMs were selected by their ability to adhere to plastic. After 1 h, non-adherent cells were removed and AMs were stimulated with LPS (10 and 100 ng/mL) for 1, 24, 48, and 72 h. Results concerning the release over time of anti-inflammatory (TGF-β) and pro-inflammatory cytokines (TNF-α and IL-6) are reported in Fig. 2. Results are expressed as the mean of a minimum of four experiments ± SEM.

Significant differences (*p* < 0.05) were observed for TNF-α and TGF-β. Although the maximum concentration of IL-6 appears at 24 h and gradually decreases in the following observation periods, no significant differences were found compared to controls.

### Characterization of the immunomodulatory effects of CM and MVs

**Fig. 3:**
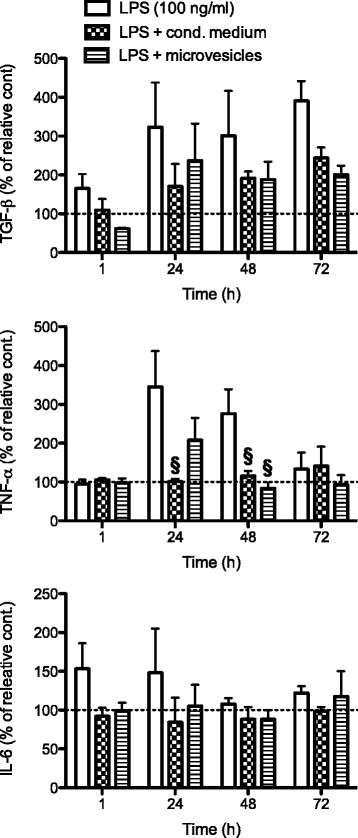
Effects of CM and MVs on the production of pro- and anti-inflammatory cytokines. Alveolar macrophages were seeded at 2 × 10^6^/mL and, after adherence, treated for 1, 24, 48, and 72 h in the presence or absence of lipopolysaccharide (*LPS*) (10 and 100 ng/mL) or of conditioned medium (*cond. medium*) or microvesicles. The release of tumor necrosis factor alpha (*TNF*-*α*), interleukin-6 (*IL*-*6*), and transforming growth factor beta (*TGF*-*β*) were evaluated in conditioned medium by ELISA. Results are the mean of a minimum of three experiments ± SEM. Statistical analysis was performed by Tukey’s multiple comparison test, with ^§^ *p* < 0.05 vs LPS-treated cells

The ability of CM and MVs to modulate LPS-induced cytokine production was then investigated. AMs were treated with LPS (100 ng/mL) in the presence or absence of CM or MVs for 1, 24, 48, and 72 h. Results (shown in Fig. 3) are presented as the mean of a minimum of three experiments ± SEM, and values expressed as a percentage of cytokine release compared to their respective control.

Significant differences (*p* < 0.05) were found for the modulatory effect of CM on LPS-induced TNF-α release at 24 h. Additionally, significant differences were observed for the modulatory capacity of CM and MVs on TNF-α release at 48 h. Furthermore, a trend toward a modulatory effect of CM and MVs on the release of TGF-β and possibly IL-6 was visible over time which, however, did not reach a statistical significance.

### Effect of TGF-β and neutralizing anti-TGF-β antibody on LPS-induced TNF-α production

**Fig. 4:**
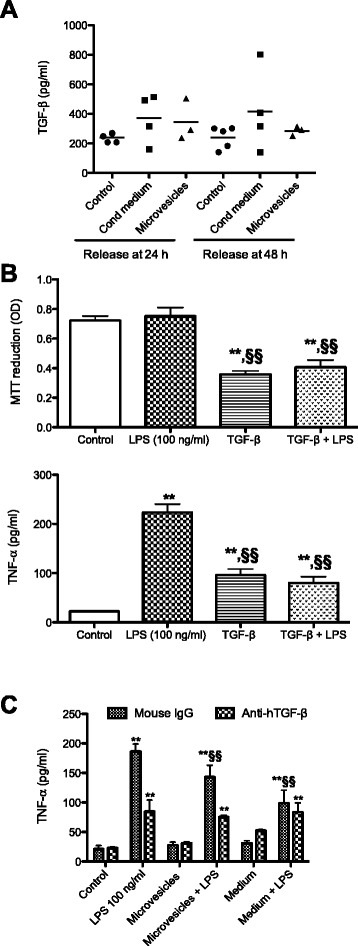
Effect of transforming growth factor beta (*TGF*-*β*) and neutralizing anti-TGF-β antibody on lipopolysaccharide (*LPS*)-induced tumor necrosis factor alpha (*TNF*-*α*) production. **a** Alveolar macrophages were seeded at 2 × 10^6^/mL and, after adherence, treated in the presence or absence of amniotic mesenchymal cell CM and MVs. Basal release of TGF-β was assessed by ELISA after 24 and 48 h of incubation. Each dot represents the value of a single animal. **b** After adherence, cells were treated in the presence or absence of equine TGF-β (300 pg/mL) and LPS (100 ng/mL) for 24 h. Cell viability was assessed by MTT test and TNF-α by ELISA. **c** After adherence, cells were treated in the presence or absence of anti-TGF-β antibody (0.2 μg/mL) or control mouse IgG (0.2 μg/mL) or CM and MVs, or LPS (100 ng/mL) for 24 h. TNF-α release was assessed by ELISA. Results are the mean of a minimum of three experiments ± SEM. Statistical analysis was performed by Tukey’s multiple comparison test, with **p < 0.01 versus relative control cells and ^§§^ *p* < 0.01 versus LPS-treated cells. *OD* optical density

In AMs treated with CM and MVs alone, a trend toward a higher release of TGF-β compared to controls was observed (Fig. 4a); at 24 h the mean values were 240 pg/mL for the control, 371 pg/mL for CM, and 343 pg/mL for MVs. Although these differences were not statistically significant (*p* > 0.05), in light of the anti-inflammatory effects of this cytokine in different experimental models the possible modulatory role of TGF-β on the observed effects was investigated. Two different experiments were set up. First, we investigated whether equine TGF-β could modulate LPS-induced TNF-α release and, second, we used a neutralizing antibody to block TGF-β. The results obtained were somewhat unexpected.

In Fig. 4b, the results concerning the effect of TGF-β on TNF-α production induced by LPS stimulation are shown. Cells were treated for 24 h in the presence or absence of TGF-β (300 pg/mL) and LPS (100 ng/mL). The concentration of TGF-β was chosen according to the mean of TGF-β measured in culture supernatants. A significant reduction (*p* < 0.01) in cell viability was observed in cells treated with TGF-β compared to controls together with a significant increase in TNF-α release (*p* < 0.01), indicative of a pro-inflammatory effect of TGF-β in AMs under resting conditions. In contrast, a statistically significant reduction in TNF-α release in cells treated with TGF-β + LPS was observed (*p* < 0.01), which may be explained by TGF-β-induced cytotoxicity.

The effects on TNF-α release when cells were treated in the presence or absence of anti-TGF-β antibody (0.2 μg/mL), control mouse IgG (0.2 μg/mL), or CM and MVs, or LPS (100 ng/mL) for 24 h are presented in Fig. 4c. The modulatory effects of CM and MVs on LPS-induced TNF-α release in the groups of cells treated with mouse IgG were confirmed. The neutralization of TGF-β resulted in a statistically significant reduction in LPS-induced TNF-α release, consistent with the pro-inflammatory effect of TGF-β observed (Fig. 4b). The release of TNF-α was not further reduced by treatment with CM and MVs. These results suggest that CM and MVs somehow act by interfering with the cellular response to TGF-β, preventing its pro-inflammatory effect.

## Discussion

Recent research focuses on strategies that allow functional restoration of damaged tissue by cell-free approaches using CM or MVs. Accordingly, this study was designed to investigate the in vitro immunomodulatory effects of CM and MVs on LPS-induced cytokine production in equine AMs, which may be beneficial in lung inflammatory disorders, including RAO. Results obtained are supportive of a potential use of CM and MVs in lung regenerative medicine, especially in situations in which TGF-β may be detrimental such as respiratory allergy. Both CM and MVs significantly reduced LPS-induced TNF-α production, together with a trend to reduction in LPS-induced TGF-β and possibly IL-6 release, with CM acting faster when compared to MVs. The latter result is consistent with the mode of action of MVs, which requires internalization, modification of gene transcription, and translation [28, 29], resulting in a delayed effect compared to CM.

It was demonstrated that AMCs secrete MVs that were able to counteract in vitro LPS-induced inflammation in tendon cells by downregulation of *MMP1*, *MMP9*, *MMP13*, and *TNFα* expression [19]. This may support the therapeutic in vivo administration of CM for the treatment of spontaneous equine tendon injuries [11] as the cargo and soluble factors of MVs could have contributed to the regenerative effect observed. MVs have been implicated in a broad, and still largely uncharacterized, range of physiological functions, such as immunity [30], signaling [28], angiogenesis, extracellular matrix (ECM) remodeling [31], and even gene regulation [32], which may also be of interest for tissue engineering. Implication of MVs in a high number of physiological functions makes them good candidates for the development of new cell-free therapies.

As TGF-β is dominantly viewed as an immune suppressive cytokine, the easiest explanation for the anti-inflammatory effects observed could have been an increase in its release by CM and MVs. However, this was not the case. On the contrary, the neutralization of TGF-β resulted in a marked reduction in LPS-induced TNF-α release, which was not due to cytotoxicity as assessed by MTT reduction, but most likely to a pro-inflammatory effect of TGF-β, supported by the ability of TGF-β alone to induce TNF-α release. TGF-β can indeed have multiple effects, resulting in both immunosuppression and immune-stimulation [33]. Our results indicate a pro-inflammatory effect in equine AMs and emphasizes the importance of the cellular and environmental context in directing the discrete role of TGF-β.

Macrophages possess unique plasticity that provides them with the ability to repair or to destroy. The repair activity is commonly associated with M2 and the destructive activity with M1 macrophages. M1 and M2 were originally defined in vivo by the preferential production of the causative functional molecules nitric oxide or ornithine, which inhibit or promote proliferation, respectively. By sensing whether to exhibit repair or destructive activities, macrophages are able to protect individuals in ways best suited to correcting non-infectious or infectious threats to hosts [34]. Their activity precedes the activation of specific immune responses and, upon encounters with M1 or M2 macrophages, other leukocytes are also induced to polarize, i.e., Th1- or Th2-types, which are characterized by the preferential production of cytokines such as IFN-γ or IL-4 that, in turn, stimulate cytotoxic T and natural killer cells, or B cells and antibody production, respectively. The clinical impact of M1 and M2 responses is therefore immense, having roles in curing or causing many diseases, including infections, cancer, autoimmunity, allergy, and atherosclerosis [34].

A predominantly M2 phenotype in equine AMs has been previously reported [35, 36]. This was also apparent in the cytokine profile we observed. While having a role in suppressing spontaneous inflammation in the lung, minimizing collateral tissue damage in a delicate tissue variably exposed to pro-inflammatory agents [37], TGF-β can have a detrimental effect in lung asthmatic reactions [34] by favoring Th2 responses and B cell antibody production. The ability of CM and MVs to contrast LPS-induced cytokine production, including TGF-β, may be beneficial in those diseases in which M2 are pathogenic. Therefore, if one has allergy, one would wish to decrease intra-lung M2-type and increase M1-type macrophages.

The cytotoxicity observed in AMs treated with TGF-β is consistent with other studies reporting the ability of TGF-β to induce apoptosis in various cell lines, particularly immune-related cells [38–41]. Even if we only used MTT reduction to assess cell viability, which does not allow us to draw any conclusions on the type of cell death (and further studies are necessary to address this point), literature evidence is supportive of apoptosis where TGF-β has been shown to regulate the mitochondria-dependent apoptotic cascade in macrophages [42, 43].

## Conclusion

Overall, results obtained showing the ability of CM and MVs to modulate equine alveolar macrophages/innate immunity are paving the way to clinical applications in equine lung diseases, such as RAO, and possibly other inflammatory disorders such as inflammatory airway disease (IAD) and exercise-induced pulmonary hemorrhage (EIPH).

## Abbreviations

AMC: Amniotic mesenchymal cell
AM: Alveolar macrophage
BALf: Bronchoalveolar lavage fluid
CM: Conditioned medium
IL: Interleukin
LPS: Lipopolysaccharide
MV: Microvesicle
RAO: Recurrent airway obstruction
TGF: Transforming growth factor
TNF: Tumor necrosis factor
